# NMR and GC/MS analysis of industrial chloroparaffin mixtures

**DOI:** 10.1007/s00216-020-02720-7

**Published:** 2020-05-29

**Authors:** Jannik Sprengel, Walter Vetter

**Affiliations:** Institute of Food Chemistry (170b), University of Hohenheim, Garbenstr. 28, 70599 Stuttgart, Germany

**Keywords:** Polychlorinated *n*-alkanes, Nuclear magnetic resonance spectroscopy, Characterization, HSQC, HMBC

## Abstract

Chlorinated paraffins (CPs) are high-volume chemicals used worldwide in various industries as plasticizers, lubricants, and flame retardants. CPs are produced by chlorination of alkane mixtures which leads to complex products of thousands of homologs and congeners. Classic mass spectrometric analyses of CPs allow determining carbon chain lengths and degrees of chlorination while information on the substitution patterns cannot be derived. Therefore, we performed different one- and two-dimensional nuclear magnetic resonance spectroscopy (NMR) experiments, elemental analysis (EA), and gas chromatography coupled with electron capture negative ion mass spectrometry (GC/ECNI-MS) for the analysis of ten technical CP products with 42%, 52%, and 70% chlorine content from four producers. Slight differences in chlorine content but varying chain length compositions were observed for similarly labeled products from different manufacturers. Two-dimensional heteronuclear spectral quantum coherence (HSQC) measurements helped to evaluate ten structural elements in the products and confirmed the presence of geminal chlorine atoms in primary and secondary carbons in products with 70% chlorine. The variation of signal groups increased with increasing chlorine content of the products. Two-dimensional heteronuclear multiple bond coherence (HMBC) analysis of one sample and GC/ECNI-MS measurements indicated the presence of impurities (e.g., C_9_-CPs, *iso*-alkanes) in different technical CP products. These methods could in future allow for better distinction of CP mixtures, and an improved trace-back of environmental CPs to the source, based on specific structural features. Additionally, further structural characterization could help in the development of more accurate analysis processes.

**Electronic supplementary material:**

The online version of this article (10.1007/s00216-020-02720-7) contains supplementary material, which is available to authorized users.

**Figure:**
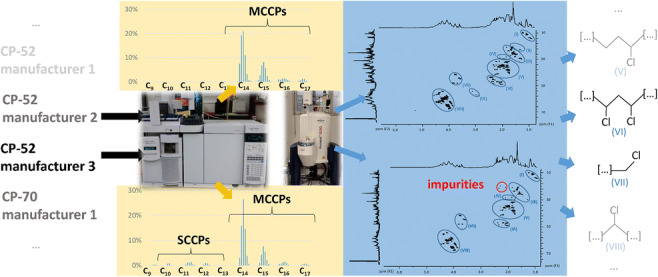
Graphical Abstract

## Introduction

Produced since the 1930s, chlorinated paraffins (CPs) are usually described as complex mixtures of polychlorinated *n*-alkanes which differ in chain length composition and degree of chlorination [1]. In China alone, more than 1 million tons of CPs is currently produced per year [2]. Applied as additives, these lipophilic and persistent chemicals can be released from products in which they are used. Due to their lipophilic and persistent nature, CPs were detected in virtually every environmental matrix, as well as food and indoor environment [3–7]. In recent years, CPs have become one of the most frequently discussed polyhalogenated, current-use pollutants worldwide [8]. From a regulatory point of view, CPs are classified according to the alkane chain length range into short-chain chlorinated paraffins (SCCPs, C_10_- to C_13_-CPs), medium-chain chlorinated paraffins (MCCPs, C_14_- to C_17_-CPs), and long-chain chlorinated paraffins (LCCPs, > C_17_-CPs) [9]. In 2017, SCCPs were classified as POPs by the Stockholm Convention [10], while no regulation has been passed for MCCPs and LCCPs. However, Chinese CP products are not primarily characterized by chain length but rather by chlorine content [2, 11].

In recent years, some studies already have examined homolog compositions (mainly SCCPs) in technical CP products from China [11–14]. Surprisingly though, little data exist about the structural composition of technical CP mixtures. Other halogenated compounds (e.g., PCBs, PBDEs) show that slight structural differences can have a great impact on their toxicity [15, 16]. Similarly, the chemical structure of halogenated compounds has an influence on their instrumental response behavior [17]. The undirected chlorination to various chlorination degrees of different paraffin (alkane) stocks leads to various, highly complex products of several thousand homologs each, which is why a better knowledge of the actual contained structural elements could greatly improve the quantification process. Additionally, impurities such as *iso*-alkanes, aromatic compounds, metals, and sulfur were reported to occur in commercial CP mixtures [18, 19]. Due to this complexity, CPs are one of the most challenging analytical problems for environmental chemists [20], and no uniform determination method could be established thus far. Instead, several quantification methods are currently applied which are almost exclusively based on mass spectrometry (MS) [21–26]. While these MS methods give insight into the chain length and the isomer distribution, further details such as structural elements (e.g., chlorine patterns of isomers) cannot be abstracted from these measurements. Some information about structural elements in technical CP mixtures can be obtained by means of nuclear magnetic resonance spectroscopy (NMR). First ^1^H NMR studies on the composition of technical CPs were performed in the 1960s and 1970s [27, 28]. However, the low magnetic field strengths of the instruments of these days and the restriction to one-dimensional ^1^H NMR experiments prohibited thorough investigations of technical CPs. Recently, modern NMR instrumentation (^1^H-NMR, ^13^C-NMR, and two-dimensional (2D) heteronuclear single quantum coherence (HSQC) experiments) was used for the analysis of several self-synthesized single-chain CP mixtures (C_10_- to C_17_-CPs, respectively) [29]. HSQC experiments show a signal for each H-X direct bond, reflecting their respective ^1^H- and (in this case) ^13^C-shifts in the *x*- and *y*-axis [30]. Therefore, these experiments were especially helpful to assign several structural elements to signal clusters [29]. Also, NMR spectra were strongly influenced by the chlorination degree of the single-chain CP mixtures, while the chain length contained could not be differentiated.

The goal in this study was to apply one- and two-dimensional heteronuclear NMR techniques (^1^H NMR, ^13^C NMR, HSQC, heteronuclear multiple bond correlation (HMBC)) to several authentic industrial CP products in order to track down characteristic patterns and substructures. We also aimed to detect impurities and wished to explore differences in industrial CPs from different producers. Complementary information was obtained from additional measurements of the industrial CP products by both gas chromatography coupled with electron capture negative ion mass spectrometry (GC/ECNI-MS) and elemental analysis. These insights might help in the categorization of CP mixtures into several groups, or even by producer, to assess possible toxicological risks posed by certain structures or impurities, and, through structural decryption, to improve quantification processes.

## Materials and methods

### Technical CP mixtures

Ten technical CP mixtures from four Chinese producers (A–D) were obtained from the respective manufacturers in 2017. The products were labeled CP-42, CP-52, or CP-70 according to their respective chlorine content (42% Cl, 52% Cl, and 70% Cl) while chain length compositions were not declared. In the following, specific technical CP products will be coded by a letter for the producer and, separated by a hyphen, a number representing the chlorine content in percent. Available products from producers A and B covered all three chlorination degrees (i.e., mixtures A-42, A-52, A-70 and B-42, B-52, B-70, respectively). Two chlorination degrees each could be analyzed from producers C and D (i.e., C-52, C-70 and D-52, D-70). The two CP-42 products were low viscous clear liquids. However, A-42 was slightly yellowish, whereas B-42 was colorless. All four CP-52 products were clear colorless liquids and slightly more viscous than the CP-42 products. In contrast to that, A-70, B-70, and C-70 were fine white powders, while D-70 was a very yellow-tinged and partly clotted powder.

### Nuclear magnetic resonance spectroscopy (NMR)

Technical CP mixtures (~ 60 mg) were dissolved in 0.7 mL deuterated chloroform (CDCl_3_, 99.8%, Deutero, Kastellaun, Germany). ^1^H and ^13^C NMR measurements of all samples were performed on a 300-MHz INOVA system (Varian, Palo Alto, CA, USA), while 2D (^1^H,^13^C)-HSQC for all samples were performed on a 600-MHz Avance III HD system (Bruker, Billerica, MA, USA). Additional ^1^H NMR spectra of samples C-52, D-52, C-70, and D-70 were recorded on the 600-MHz instrument. Parameters for the ^1^H and ^13^C NMR as well as the HSQC measurements were described elsewhere [29]. An additional (^1^H,^13^C)-HMBC experiment of sample D-52 was performed on the 600-MHz instrument using acquisition times of 0.19 s (F2) and 0.03 s (F1), a spectral width of 5400 Hz (F2) and 18,100 Hz (F1), and 2048 × 1024 data points over 96 scans (run time ~ 60 h). Chemical shifts were referenced to the signal of residual CHCl_3_ in the solvent CDCl_3_ at δ(^1^H) = 7.26 ppm and δ(^13^C) = 77 ppm. NMR data was processed using SpinWorks 4.0.5 software (University of Manitoba, Winnipeg, Canada, 2014). Additional experiments (DEPT-90, DEPT-135, decoupled ^1^H NMR, ^1^H NMR in C_6_D_6_) were performed on some samples, but resulted in unserviceable spectra (see Electronic Supplementary Material (ESM) Figs. S9–S11).

### Elemental analysis (EA)

The carbon content of the technical CP mixtures (~ 1.5 mg in 99.9% Sn capsules, Hekatech, Wegberg, Germany) was determined on a Euro EA 3000 elemental analyzer (Hekatech, Wegberg, Germany) in combination with a Delta plus XP system (Thermo Finnigan MAT, Bremen, Germany) with conditions described in a previous publication [31]. For hydrogen measurements, the elemental analyzer was combined with a high-temperature oven equipped with a self-developed pyrolysis reactor (Hekatech, Wegberg, Germany) and a chromium reduction reactor [32]. Ca. 1.5 mg of the technical CP products was weighed into silver capsules (99.9% Ag, Hekatech, Wegberg, Germany).

### Gas chromatography coupled with electron capture negative ion mass spectrometry (GC/ECNI-MS)

Dilutions of all technical CP mixtures (~ 120 ng/μL in *iso*-octane, *for pesticide residue analysis*, Fluka Analytics, Seelze, Germany) were measured in full scan mode (*m*/*z* 50–800) on a 7890/5975C MSD system (Agilent, Waldbronn, Germany) equipped with an OPTIMA-5 MS column (30 m × 0.25 mm i.d., 0.25 μm *d*_f_; Macherey-Nagel, Düren, Germany). Instrumental parameters were described elsewhere in details [5]. The CP homolog composition was determined by means of the most abundant isotope peaks of the [M-Cl]^−^ ions in full-scan chromatograms for all CPs with 9 to 17 carbon atoms and 4 to 12 chlorine atoms (Section S2 in ESM).

## Results and discussion

### GC/ECNI-MS determination of chain length distribution and chlorine content of technical CP products

**Fig. 1:**
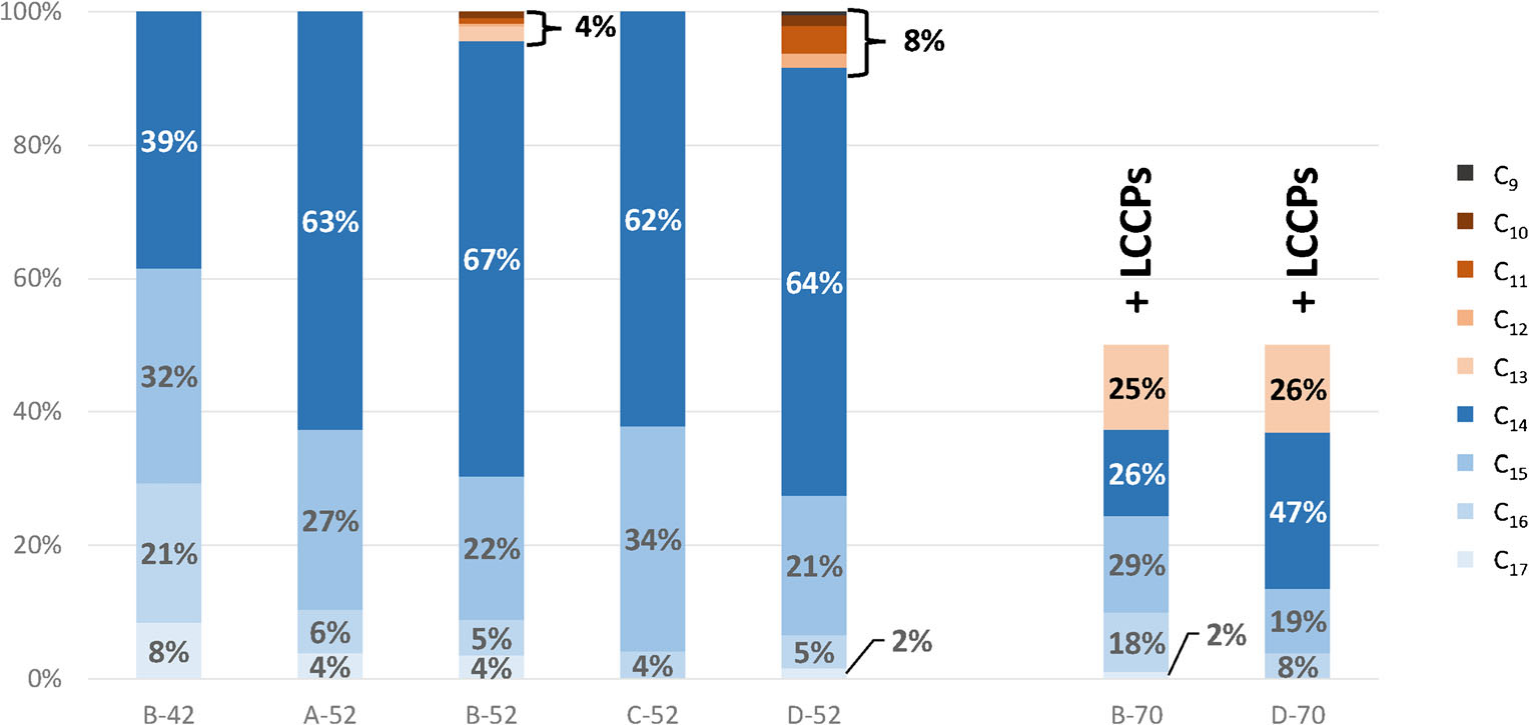
Relative chain length distribution of the technical CP products A-52, B-42, B52, C-52, and D-52 as determined by GC/ECNI-MS. Additionally, the chain distribution of the MCCP portion of B-70 and D-70 is shown. It has to be considered, however, that these two samples mainly consisted of (not detectible) LCCPs

Five mixtures (A-/B-/C-/D-52 and B-42) mainly consisted of MCCPs (Table 1). Only samples B-52 and D-52 also contained ~ 4% and ~ 8% SCCPs. These five MCCP products were dominated by C_14_-CPs (38.5–66.6%), which is in accordance with the general prevalence of C_14_-CPs among MCCPs in the environment [12], CP containing products [33], and a previously analyzed technical CP-52 product [34]. In addition, C_15_-CPs > C_16_-CPs > C_17_-CPs were detected with decreasing shares. The CP-52 products showed rather similar chain length compositions, but B-42 differed considerably from the other products because it contained much higher shares of C_16_- and C_17_-CPs (Fig. 1). A recent study reported highly varying chain length compositions in commercial CP products from China [11]. The technical CP-42 and CP-52 products analyzed in that study were dominated by C_13_-CPs, which was different to our samples (see above). This could indicate a shift to MCCP production in order to omit the presence of SCCPs following their classification as POPs in 2017. All CP-70 samples showed a single large hump of relatively low intensity (ESM Fig. S1). In samples B-70 and D-70, mass spectra indicated the presence of highly chlorinated (> Cl_10_) C_13_- to C_17_-CPs, which were strongly overlapped by other high mass signals (mainly *m*/*z* > 500, ESM Fig. S2). This, together with the very low intensity of the signals, indicated that these samples were mainly composed of LCCPs, which could not be adequately measured by GC/MS due to their low volatility [35, 36].

**Table 1:** Product type (SCCP, MCCP, LCCP), appearance, chlorine content (as determined by EA), and identified impurities of the ten analyzed technical CP products. Additionally, a mean carbon formula (CH_*x*_Cl_*y*_), indicating the approximate mean number of chlorine/hydrogen atoms per carbon atom, is given. The area of the “monochlorinated” range in the ^1^H NMR spectrum (section “^1^H and ^13^C NMR spectra”) of each sample was given relative to the normalized area (1.000) of the “unchlorinated” range

Sample	Product type	Appearance	Cl content [%]	Relative area of the “monochlorinated” range in ^1^H NMR	Mean carbon formula (CH_*x*_Cl_*y*_)	Impurities
A-42	LCCP	Clear, yellowish liquid	41.1 ± 0.3	0.184	CH_1.86_Cl_0.26_	–
B-42	MCCP	Clear colorless liquid	46.5 ± 2.7	0.235	CH_1.75_Cl_0.37_	–
A-52	MCCP	Clear colorless liquid	53.1 ± 0.2	0.301	CH_1.68_Cl_0.44_	–
B-52	MCCP (+SCCP)	Clear colorless liquid	52.3 ± 0.8	0.322	CH_1.68_Cl_0.44_	SCCPs
C-52	MCCP	Clear colorless liquid	53.0 ± 0.7	0.326	CH_1.68_Cl_0.45_	–
D-52	MCCP (+SCCP)	Clear colorless liquid	53.6 ± 0.8	0.303	CH_1.67_Cl_0.46_	SCCPs + C_9_-CPs + aromatics
A-70	LCCP	Fine white powder	71.7 ± 0.1	1.065	CH_1.20_Cl_0.93_	*n*-Alkanes
B-70	MCCP/LCCP (+SCCP)	Fine white powder	73.8 ± 0.1	1.606	CH_1.12_Cl_1.00_	*n*-Alkanes + *iso*-alkanes
C-70	LCCP	Fine white powder	69.5 ± 0.1	0.908	CH_1.30_Cl_0.82_	Halogenated aromatics
D-70	MCCP/LCCP	Yellow, partly clotted powder	70.6 ± 0.4	1.053	CH_1.27_Cl_0.85_	Halogenated aromatics

GC/ECNI-MS measurements of samples A-42, A-70, and C-70 did not indicate measurable amounts of SCCPs or MCCPs. Their GC/MS chromatograms likewise showed a single large hump, while the mass spectra were comprised of overlapping high mass signals with no discernible signals for SCCPs or MCCPs (ESM Fig. S2**)**. Therefore, these samples most likely exclusively consisted of LCCPs. Accordingly, Li et al. found high amounts of LCCPs in two out of three CP-70 mixtures using UPLC-ESI-QTOFMS [11]. Unfortunately, insufficient response from higher chlorinated homologs or longer chain lengths [35] heavily impeded further characterization of these products by GC/MS. Interestingly, LCCP-containing A-42 had a yellowish tinge in contrast to B-42, which did not contain LCCPs and was a clear liquid. This was confirmed by spectrophotometric color measurements, which yielded lower lightness (*L*) and slightly higher values for yellowish color (*b*) (ESM Section S1, Table S1).

Apart from the presence of SCCPs (C_10_–C_13_ alkanes, section “GC/ECNI-MS determination of chain length distribution and chlorine content of technical CP products”), GC/ECNI-MS analysis of sample D-52 also enabled the detection of trace amounts (< 1%) of C_9_-CPs (ESM Fig. S7). C_9_-CPs do not belong to the class of SCCPs. Instead, they were classified as “very short chain chlorinated paraffins” (vSCCPs), which are not analyzed very often [37]. However, Zhou et al. and Xia et al. also detected traces of C_9_-CPs in overall five technical CP-52 products from China [37, 38].

EA measurements of the technical CP products (section “Elemental analysis (EA)”, ESM Table S3) resulted in similar but not identical chlorine contents as listed on the corresponding labels (42%, 52%, and 70% Cl, respectively, Table 1). Only samples B-42 (46.5% Cl) and B-70 (73.8% Cl) differed by more than 2% Cl from the specified value. Chlorine, carbon, and hydrogen contents were used to calculate a “mean carbon formula” (Section S3 in ESM), which expresses the mean number of chlorine and hydrogen atoms per carbon (e.g., CH_1.86_Cl_0.26_ for A-42, Table 1). Accordingly, CP-42 products had on average one Cl substituent on every third carbon atom, CP-52 products one Cl substituent on every second carbon, and CP-70 products one Cl substituent on almost every carbon atom (Table 1). However, slight differences in the actual chlorine content per weight could cause a difference of up to ~ 30% in the average number of chlorine atoms per carbon atom for CP products of the same chlorination degree (Table 1). These findings were taken into account during the NMR analyses.

### Nuclear magnetic resonance spectroscopy (NMR) analysis of technical CP products

#### ^1^H and ^13^C NMR spectra

**Fig. 2:**
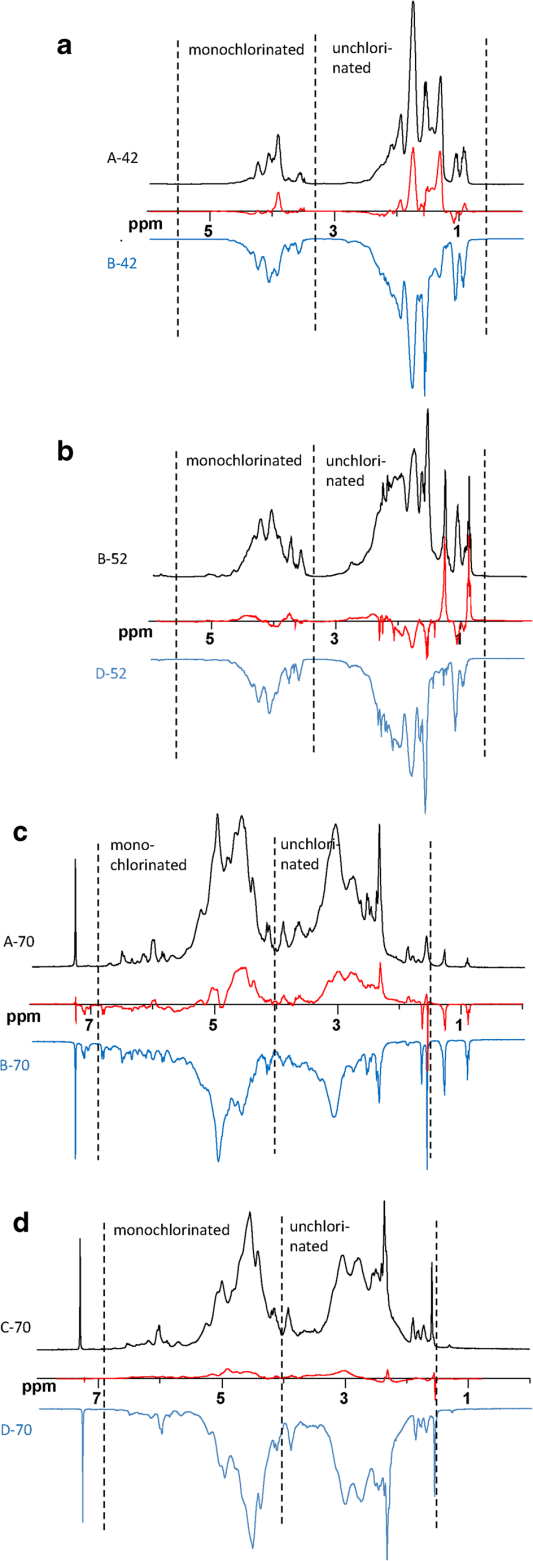
^1^H NMR spectra (300 MHz) of **a** two CP-42 products, **b** B-52 (A-52 being nearly identical) and D-52 (C-52 being nearly identical), and **c + d** four CP-70 products. The subtracted spectra are displayed in red on the *x*-axis

^1^H and ^13^C NMR spectra of all ten technical CP mixtures showed the known unresolved clusters of multiplets [27–29] (Fig. 2, ESM Figs. S3 and S4). Similar to single-chain length CP standards, CP-42 and CP-52 products showed two distinct clusters, i.e., the “unchlorinated” range (δ(^1^H) = 0.8–3.2 ppm; δ(^13^C) = 15–50 ppm) and the “monochlorinated” range (δ(^1^H) = 3.2–5.3 ppm; δ(^13^C) = 54–72 ppm) [29]. At a stronger field (600 instead of 300 MHz), both ranges could not be further resolved into smaller subclusters. In the ^1^H NMR spectra of the four CP-70 products, the clusters of both the “unchlorinated” range and “monochlorinated” range in ^1^H NMR spectra were shifted by ~ 0.7 ppm further downfield, i.e., 1.5–4.0 ppm and 4.0–6.8 ppm (Fig. 2c, d). In addition, the stretch of the “monochlorinated” range was also increased from 2.1 to 2.8 ppm in CP-70 products. After an acceptable measuring time, ^13^C NMR spectra showed only noisy signals and could not be evaluated in a meaningful way. Yet, it was observed that the relative abundance of the “unchlorinated” range ^13^C-cluster at δ(^13^C) = 15–50 ppm decreased with increasing chlorine content.

##### Technical CP-52 products

^1^H NMR spectra of the four CP-52 products were characterized by very similar shapes of the “monochlorinated” range (Fig. 2b). In the “unchlorinated” range, products A-52 and B-52 gave elevated signal intensities at δ(^1^H) = 1.0–1.2 ppm and 1.4–1.5 ppm compared to C-52 and D-52, which was confirmed by spectrum subtraction of B-52 and D-52 (see subtracted spectra of B-52 and D-52 in Fig. 2b, red line). These subtracted spectra show subtle differences between the samples that would not be readily visible from their original spectra. This indicated higher amounts of [CH_3_–CH_2_–] moieties in A-52 and B-52 compared to C-52 and D-52. In contrast, the signal range at δ(^1^H) = 1.4–2.1 ppm was more pronounced in sample D-52.

##### Technical CP-42 products

In the “monochlorinated range,” the highest signal abundance in the ^1^H NMR spectrum of A-42 was slightly shifted to a higher field (δ(^1^H) = 4.0–4.15 ppm) compared to B-42 (δ(^1^H) = 4.15–4.25 ppm, Fig. 2a). Moreover, the abundance at δ(^1^H) > 4.5 ppm was also higher in B-42. Both observations were in agreement with the higher chlorine content of B-42 by ~ 5% Cl (+ Cl_0.11_ (~ 30%) per carbon) as determined by EA (Table 1). The higher chlorine content of B-42 also caused a shift in the signal intensities in the “unchlorinated range.” According to the subtracted ^1^H NMR spectra, B-42 featured more abundant signals at δ(^1^H) = 2.0–2.5 ppm and 4.1–4.5 ppm, while signals at 1.3–1.8 and 3.8–4.0 were more abundant in A-42 (Fig. 2a, red line).

##### Technical CP-70 products

Based on the mean carbon formula, A-70 and B-70 (0.93 and 1.0 Cl/carbon) were richer in Cl than C-70 and D-70 (0.82 and 0.85 Cl/carbon, Table 1). The difference between B-70 and C-70 was about one additional Cl per five carbons in B-70, which is considerably high. Accordingly, the ^1^H NMR spectra of C-70 and D-70 were almost identical as can be seen in the subtracted spectrum (Fig. 2d, red line). The rather similar Cl number of C-70 and D-70 was in agreement with the very similar ^1^H NMR spectra of both products. By contrast, ^1^H NMR spectra of A-70 and B-70 strongly differed from those of the other samples (Fig. 2c, d), but also from each other (Fig. 2c, red line). Furthermore, the higher mean Cl number of A-70 and B-70 was reflected in a downfield shift of the monochlorinated range of these two samples (compare Fig. 2c and d). For a closer inspection, all CP samples were analyzed by HSQC.

#### HSQC measurements of ten technical CP mixtures

**Fig. 3:**
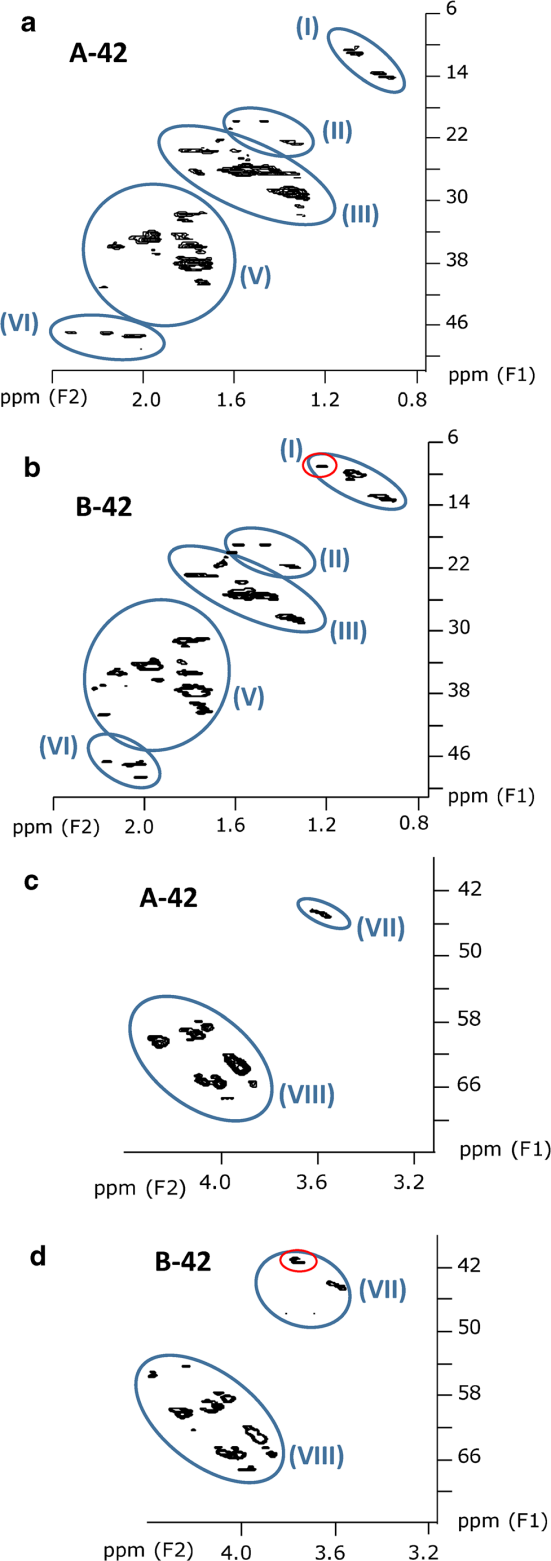
HSQC spectra excerpts (600 MHz) of samples **a + c** A-42 and **b + d** B-42. These excerpts show all signals (except solvent residue) found for these samples. The signals marked by a red circle indicate a higher abundance of methyl groups in B-42 (MCCPs) compared to A-42 (LCCPs), reflecting the difference in chain length

**Fig. 4:**
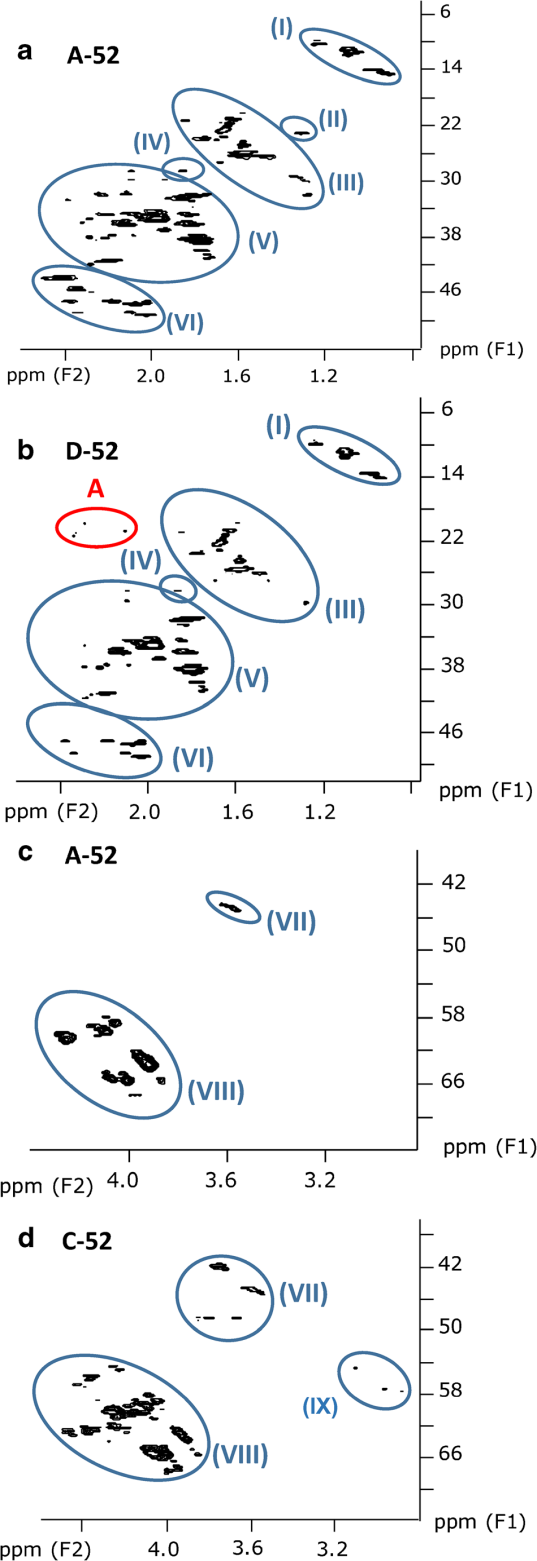
HSQC spectra excerpts (600 MHz) of samples **a + c** A-52, **b** D-52, and **d** C-52. While **a** and **c** showcase the general signal distribution found in HSQC spectra of CP-52 products, **b** and **d** illustrate all unusual signal appearances in those samples. Signal group “A” could not be identified, but probably originated from impurities rather than from the CPs themselves

Based on previous assignments in single-chain CP mixtures [29], ten subclusters could be defined in the HSQC spectra of the commercial CP products (Table 2, Fig. 3 and 4). Subsequently, the corresponding subclusters were classified according to their intensity. In HSQC spectra, the signal intensity is visualized by size which corresponds with the ppm range on both axes. Within a cluster, individual signals with dimensions of > 0.2 ppm (F2) and > 2 ppm (F1) were defined as abundant signals. For each cluster, four scenarios were defined based on the number and intensity of the individual signals, i.e.,

- +: < 4 signals and max. 1 abundant signal
- ++: > 4 signals but max. 2 abundant signals
- +++: > 4 signals and 3–4 abundant signals
- ++++: > 4 abundant signals

**Table 2:** Qualitative subcluster profiles in technical CP mixtures. Signals with ranges > 0.2 ppm for ^1^H or > 2 ppm for ^13^C were defined as abundant signals

Subcluster		Sample									
No.	Structure	A-42	B-42	A-52	B-52	C-52	D-52	A-70	B-70	C-70	D-70
(I)	**CH**_**3**_**–**CH_2_–	++	++	++	++	++	++				
(II)	–CH_2_**–CH**_**2**_**–**CH_2_–	++	++	+	+	+					
(III)	**CH**_**3**_**–**CHCl–	++++	+++	+++	+++	+++	++	+		++	++
(IV)	Unknown			+	+	+	+				
(V)	–CH_2_**–CH**_**2**_**–**CHCl–	++++	++++	++++	++++	++++	++++	++	++	++	++
(VI)	–CHCl**–CH**_**2**_**–**CHCl–	++	++	+++	+++	+++	+++	+++	++	++++	++
(VII)	**CH**_**2**_**Cl–**	+	++	++	++	++	++	++	++	++	++
(VIII)	**–CHCl–**	++++	++++	++++	++++	++++	++++	++++	++++	++++	++++
(IX)	–CCl_2_**–CH**_**2**_–CHCl–					+		+++	++	+++	+
(X)	**CHCl**_**2**_–							++	++	++	++

+ < 4 and max. 1 abundant signal, ++ max. 2 abundant signals, +++ 3–4 abundant signals, ++++ > 4 abundant signals

This classification scheme was used for a semi-quantitative comparison of subcluster profiles in the different technical CP products (Table 2). Subclusters (I)–(III) and (V) (all associated with low chlorinated substructures) showed a significant (*p* < 0.05) negative linear correlation with the chlorine content, while subcluster (X) (**CHCl**_**2**_**–**) was significantly positively correlated with the chlorine content (ESM Table S2). These results were in accordance with our previous findings with single-chain CP mixtures [29].

Similar to the ^1^H NMR spectra, HSQC subcluster profiles of the CP-52 mixtures differed only marginally (Table 2). Only slight differences in one of these products were observed with regard to the occurrence or intensities of subclusters (II) and (III) (Table 2, Fig. 4b). For instance, the [–CH_2_–**CH**_**2**_**–**CH_2_–] signal (subcluster (II)) was missing in sample D-52. Likewise, only C-52 which contained the lowest share of longer-chain MCCPs (~ 4%, compared to 7–10%; Fig. 1) featured the –[–CCl_2_**–CH**_**2**_**–**CHCl–] subcluster (IX). This indicated that not only Cl% but also the synthesis conditions had an impact on the occurrence of substructures.

It is noteworthy that all four CP-52 products and only these featured the small subcluster (IV) corresponding with ~ 2 ppm in the ^1^H NMR and ~ 30 ppm in the ^13^C spectrum, which is about the center of the unchlorinated range (Table 2, ESM Fig. S6). This signal was also present in two C_11_-CP mixtures, one with about the same chlorine content (51.2%) and one with a higher chlorine content (62.6%) [29]. This could be indicative that this subcluster is rather characteristic of CP mixtures with intermediary Cl content (50–65%) that are dominated by SCCPs/MCCPs. The only MCCP product not featuring (IV) was B-42, which had a much lower chlorine content than the other SCCP/MCCP samples analyzed.

Technical CP-52 products also differed from CP-42 products by a higher abundance of the [–CHCl**–CH**_**2**_**–**CHCl–] subcluster (VI). At the same time, [–CH_2_**–CH**_**2**_**–**CH_2_–] subcluster (II) decreased from medium (++) in CP-42 to low (+) intensity in CP-52 samples. These observations were in accordance with the higher chlorine density in CP-52 mixtures. CP-52 products had a mean carbon formula of CH_1.68_Cl_0.45_ (Table 1). Hence, about every second carbon carried a chlorine substituent (considering that primary carbons are scarcely chlorinated at intermediate chlorine content). Therefore, [–CH_2_**–CH**_**2**_**–**CH_2_–] moieties (subcluster (II)), indicative of three subsequent carbons without Cl, were scarcely found.

The different chain length distributions of A-42 and B-42 (section “GC/ECNI-MS determination of chain length distribution and chlorine content of technical CP products”) and different chlorine densities (0.26 vs. 0.37 Cl per carbon, Table 1) were not readily visible in the HSQC spectra (Fig. 3). However, subclusters (III) and (VII) both showed additional signals in the upper-left range (i.e., regions downfield in ^1^H and highfield in ^13^C NMR), with subcluster (VII) featuring two extra signals (Fig. 3c). This may indicate a higher share of methyl groups as well as some chlorine atoms located on terminal carbon atoms in B-42. This interpretation was in agreement with the shorter alkyl chains and higher Cl content of B-42.

**Fig. 5:**
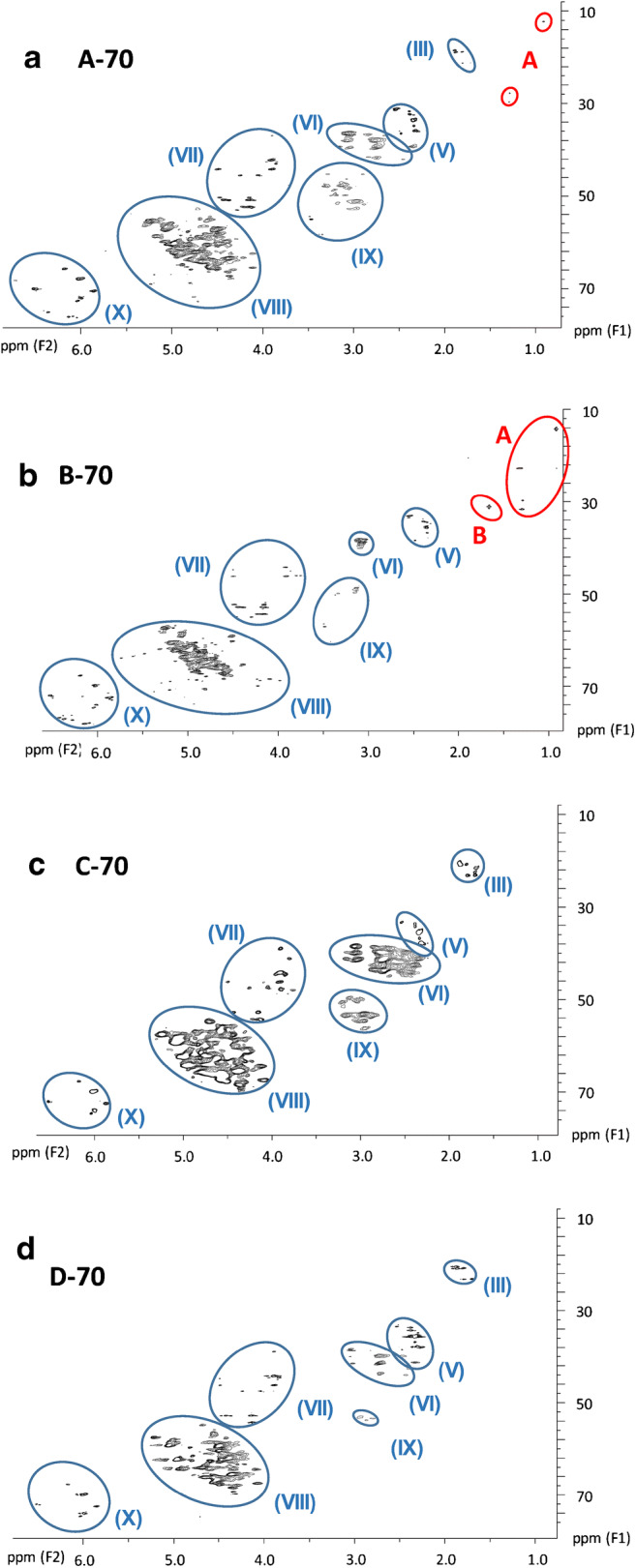
HSQC spectra (600 MHz) of samples **a** A-70, **b** B-70, **c** C-70, and **d** D-70. Signal group “A” likely originated from solvent impurities in the product, while signal group “B” could originate from branched alkanes. The large area differences between A-70 and B-70 mainly originated from the lower hydrogen content of B-70 by ~ 10% (relative to A-70)

Similar to ^1^H NMR measurements (section “^1^H and ^13^C NMR spectra”), the HSQC spectra of the four CP-70 mixtures differed pronouncedly in their subcluster profiles (e.g., A-70 and C-70, Table 2, Fig. 5a, c). For example, sample B-70, having the highest chlorine content of all measured CP products, was missing any signals in subcluster (III), indicating that unchlorinated chain ends did not exist in this product. The difference between the ^1^H NMR spectra of A-70 and B-70 (section “^1^H and ^13^C NMR spectra”) could probably be explained by the HSQC patterns as well, as A-70 showed more signals of unchlorinated carbon atoms in chlorinated neighborhoods (e.g., subclusters (III), (VI), and (IX)). Interestingly, C-70 and D-70 whose ^1^H NMR spectra were very similar (section “^1^H and ^13^C NMR spectra”) showed remarkable differences in the HSQC substructure profile. Namely, subclusters (VI) and (IX), both originating from protons of non-chlorinated carbons but with adjacent chlorine substituents, were larger in C-70 (Table 2). However, the ^1^H shift range of subclusters (VI) and (IX) was strongly overlapping and the main difference was in the ^13^C shifts with (VI) < (IX) (Fig. 5c, d). Considering that D-70 contained shorter alkane chains, there are fewer possibilities for chlorine-free carbon atoms, which might lead to the decrease of signals in those two subclusters.

In summary, no two technical CPs of the same type were completely identical. The biggest similarities were found between NMR spectra of CP-52 products; however, even those differed in chain length composition (section “GC/ECNI-MS determination of chain length distribution and chlorine content of technical CP products”). CP-42 and CP-70 products showed a much greater variance not only in chain length distribution but also in chlorination degree. At this point, it should be recalled that all CP-70 as well as A-42 consisted of LCCPs (section “GC/ECNI-MS determination of chain length distribution and chlorine content of technical CP products”). These largely unknown LCCP products were probably more affected by different alkane compositions and reaction conditions.

### Geminal chlorine substituents ([–CCl_2_–] moieties) in technical CP products

It has been mentioned that [–CCl_2_–] moieties are unlikely to be formed during technical CP synthesis [20]. However, the high chlorine density of CP-70 products with 0.82–1.0 Cl substituent per carbon atom produced strong evidence that such moieties should exist. Unfortunately, [–CCl_2_–] moieties give no signal in HSQC spectra. Therefore, their direct presence had to be verified by means of ^13^C NMR in the form of resonances at δ(^13^C) = 85–98 ppm [29]. Despite the generally low sensitivity of ^13^C NMR, signals at δ(^13^C) = 85–98 ppm were clearly detected in all CP-70 products (ESM Fig. S4). Moreover, all technical CP-70 products showed HSQC signals corresponding with [–CCl_2_–CH_2_–CHCl–]– and [CHCl_2_–] groups (subclusters (IX) and (X), Fig. 5).

**Fig. 6:**
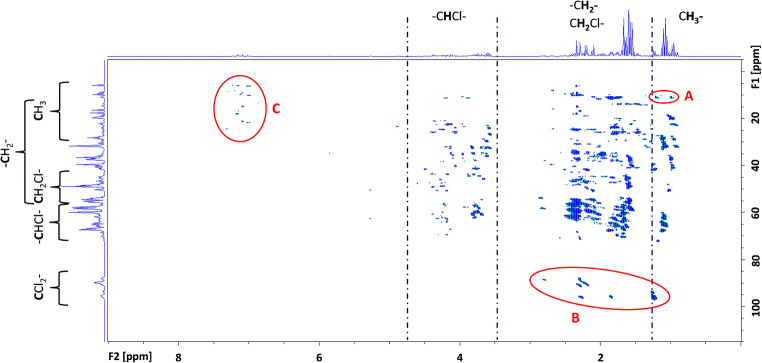
HMBC spectrum (600 MHz) of sample D-52. The shift ranges for certain substructures are indicated on the F1/F2 traces. Signal groups “A” and “B” pointed to (chlorinated) *iso*-alkanes and dichlorinated carbon atoms (–CCl_2_–) being present in this sample, respectively, while signal group “C” (aromatics) likely originated from impurities in the CP mixture

Interestingly, the (only recorded) HMBC spectrum (measuring time ~ 60 h) of sample D-52 showed peaks in the range δ(^13^C) = 85–98 ppm (Fig. 6). In an HMBC experiment, H–X couplings are illustrated similarly to the HSQC, but over multiple (2–3) bonds instead of direct bonds [30]. The corresponding ^1^H signals indicated neighbored hydrogens in (unchlorinated) methylene groups separated by two bonds (Fig. 6). These signals contributed with ~ 6% to the total signal area of signals in the HMBC spectrum. Therefore, even at a medium chlorination degree, dichlorinated carbon atoms may occur in technical CP products. This is important because [–CCl_2_–] moieties are likely those that can be transformed most easily by reductive dechlorination.

### Impurities in CP products

GC/ECNI-MS chromatograms of samples C-70 and D-70 featured single peaks of two halogenated compounds at 17.61 and 18.50 min (i.e., prior to the CP hump). The corresponding mass spectra indicated the presence of [M−Cl]^−^ fragment ions of hexachlorinated compounds (ESM Fig. S8). Based on [M−Cl]^−^ at *m*/*z* 273 (ESM Fig. S8a), peak 1 corresponded with the molecular formula C_8_H_2_Cl_6_ which could be a hexachlorostyrene isomer. It is noteworthy that impurities in the form of aromatic compounds were previously described in the literature [18, 19]. Interestingly, the HMBC spectrum of sample D-52 showed a signal group at δ(^1^H) = 7.0–7.4 ppm and δ(^13^C) = 8–24 ppm, which also indicated the presence of aromatic compounds with aliphatic side chain(s). However, polyhalogenated aromatic compounds could not be detected by GC/ECNI-MS measurements of D-52. Hence, this signal group could originate from low- or non-halogenated aromatic compounds. The peak area of these impurities in the technical CP product contributed with ~ 1.5% of the signals in the HMBC spectrum of D-52.

In addition, a small signal group in the HMBC spectrum of sample D-52 (0.5% of the total area) was detected at δ (^1^H) = 1.1–1.3 ppm and δ(^13^C) = 10–14 ppm. Both shifts are typical for secondary methyl groups, which indicated the presence of branched CPs in D-52. In accordance, *iso*-alkanes were reported to exist in technical CP products [18, 39]. Unfortunately, recording of the HMBC spectrum was very time consuming (~ 60 h) and could not be carried out with all technical CP products. However, the benefits of this method are obvious.

B-70 and, to a lesser extent, A-70 showed HSQC signals at δ(^1^H) = 0.88–0.89 ppm and 1.26 ppm **(**Fig. 2c). These highfield signals indicated the presence of non-halogenated *n*-alkanes (e.g., *n*-hexane or mineral oils) in the two CP-70 products [40]. However, if these alkanes had been present during the synthesis, they would have been chlorinated as well. Therefore, these impurities were likely introduced after the chlorination was completed. Based on the peak areas in the ^1^H NMR spectra, these residues contributed < 1% (w/w) to both technical products. Sample B-70 showed additional HSQC signals at δ(^1^H) = 1.63 ppm and δ(^13^C) = 30–34 ppm, which did not appear in other technical CP-70 products (Fig. 5a, b, in signal group “B”). In HSQC spectra, the corresponding signals were found at the fringe of subcluster (III) [**CH**_**3**_**–**CHCl–]. However, tertiary carbon atoms of branched alkanes show shifts in a similar range (δ(^1^H) = 1.56 ppm and δ(^13^C) = 28.1 ppm) [41]. Therefore, these signals in B-70 (and possibly a small part of the signals of subcluster (III) in CP-42 and CP-52 products) most likely originated from *iso-*alkanes, too.

## Conclusions

It could be demonstrated that data obtained from NMR and especially HSQC measurements allows the study and differentiation of technical CP products. The results are supplementary to those obtainable by GC/MS and LC/MS methods. The combination of analytical methods helps to understand the structural elements of technical CP products and provides a groundwork for further structural analyses. In the future, such multi-method approaches may also help to categorize CP products and trace back environmental CP contaminations to the source. Structural knowledge may also greatly assist in the development of more precise quantification methods. Likewise, the direct detection of impurities in five of ten CP products is worth noting. The occurrence of aromatic and branched compounds in CP products should be considered in future toxicological studies of CPs.

## Electronic supplementary material

ESM 1 (PDF 1228 kb).
