# Supplementary material for: NMR and GC/MS analysis of industrial chloroparaffin mixtures
Source: Anal Bioanal Chem. 2020 May 29;412(19):4669–79. doi: 10.1007/s00216-020-02720-7 (PMC7329780; doi:10.1007/s00216-020-02720-7)
Supplement: Supplementary file 1 — (PDF 1228 kb). [file 216_2020_2720_MOESM1_ESM.pdf]

Analytical and Bioanalytical Chemistry

Electronic Supplementary Material

NMR and GC/MS analysis of industrial chloroparaffin mixtures

Jannik Sprengel, Walter Vetter

S1: *Color measurements.*

Color measurements were performed with a CM-700d spectrophotometer (Konica Minolta, Tokyo, Japan). Ca. 200 µL of the liquid CP mixture was applied on a deepening of a clear plastic surface, which was placed on a white sheet of paper. After "White Calibration" of the instrument against the White Calibration Cap (CM-A177), the measuring port of the instrument was placed directly on the plastic surface. Mean values of five measurements for each of the samples were calculated in the program Color Data Software CM-S100w SpectraMagic NX Lite Version 2.7 (Konica Minolta, Tokyo, Japan) (Tab. S1).

S2: *Homolog composition*

The homolog composition of the technical CP products was calculated from the chlorine number normalized relative areas of the most abundant isotope of the $[M\text{-}Cl]^-$-cluster from Cl_4- to Cl_{10}-homologs (equation 1).

$$[\%]x = \frac{\frac{A_x}{Cl_x}}{\sum \frac{A_x}{Cl_x}} \qquad (1)$$

[%]x = share of the homolog x to the sum of CPs

A_x = peak area of the most abundant isotope of the [M-Cl[- cluster of homolog x

Cl_x = number of chlorine atoms in homolog x

$\sum A_x$ = sum peak area of all homologs measured

S3: *Mean single carbon atom formula*

The "mean single carbon atom formula" was calculated from the determined carbon content analogous to the procedure demonstrated in a previous publication [1]. The average number of heteroatoms per carbon atom changes ~6% from C_{10}- (2.20 heteroatoms) to C_{30}-alkane bodies (2.07 heteroatoms). For the unknown mixtures, the value for hexadecane (2.13 heteroatoms) was used as an approximation, as this value represented the mean value between the two extreme values.

$$y = \frac{M(C)\cdot(100-\%(C))}{\%(C)\cdot(M(Cl)-M(H))} - \frac{2.125\cdot M(H)}{M(Cl)-M(H)} \qquad (2)$$

y = average number of Cl atoms per carbon atom

M(Cl) = atomic mass of chlorine (35.453 u)

M(H) = atomic mass of hydrogen (1.0079 u)

M(C) = atomic mass of carbon (12.0107 u)

%(Cl) = chlorine content by mass [g/g = %]

Tables

Table S1 Values for lightness (L*), greenish-reddish color (a*, a*<0 = greenish colors and a*>0 = reddish colors) and bluish-reddish color (b*; *, b*<0 = bluish colors and b*>0 = yellowish colors) of color measurements of technical CP products A-42 and B-42. The mean value of five measurements each is given. The slight bluish background and relatively high lightness value probably originated from the sheet of paper used as background [2]

	L*	a*	b*
**A-42**	44.53	0.61	2.21
**B-42**	57.88	0.97	-1.65
**Δ**	13.35	0.36	-3.86

Table S2 Pearson coefficients and correlation significance between the strength of signal groups (I) - (X) and the chlorination degree. Signal groups with strong and significant correlations are marked in bold

Signal group	Pearson coefficient	significance
**(I)**	**-0.943**	**0.036**
**(II)**	**-0.873**	**0.047**
**(III)**	**-0.878**	**0.046**
(IV)	-0.404	0.290
**(V)**	**-0.943**	**0.036**
(VI)	0.183	0.619
(VII)	0.521	0.186
(VIII)	-	-
(IX)	0.830	0.055
**(X)**	**0.943**	**0.036**

Table S3 Hydrogen and carbon content of ten technical CP products as determined by EA-IRMS

Sample	H-content [%]	C-content [%]
A-42	7.0 ± 0.1	51.9 ± 0.2
B-42	8.9 ± 0.1	44.6 ± 2.6
A-52	6.0 ± 0.1	41.0 ± 0.1
B-52	6.9 ± 0.0	40.8 ± 0.8
C-52	6.4 ± 0.1	40.6 ± 0.6
D-52	6.3 ± 0.1	40.2 ± 0.7
A-70	2.2 ± 0.1	26.1 ± 0.0
B-70	1.6 ± 0.0	24.6 ± 0.1
C-70	2.2 ± 0.1	28.2 ± 0.0
D-70	1.8 ± 0.2	27.6 ± 0.2

Table S4 Similarities between the compositions of ten technical CP products. Samples marked with an asterisk(*) were mainly comprised of LCCPs, therefore the chain distribution

Sample	chain distribution similar to	^{1}H NMR spectrum similar to	HSQC spectrum similar to
A-42	-	(B-42)	(B-42)
B-42	-	(A-42)	(A-42)
A-52	other CP-52	other CP-52	other CP-52
B-52	other CP-52	other CP-52	other CP-52
C-52	other CP-52	other CP-52	other CP-52
D-52	other CP-52	other CP-52	other CP-52
A-70	*C-70?	-	C-70
B-70	*?	-	D-70
C-70	*A-70?	D-70	A-70
D-70	*?	C-70	B-70

Figures

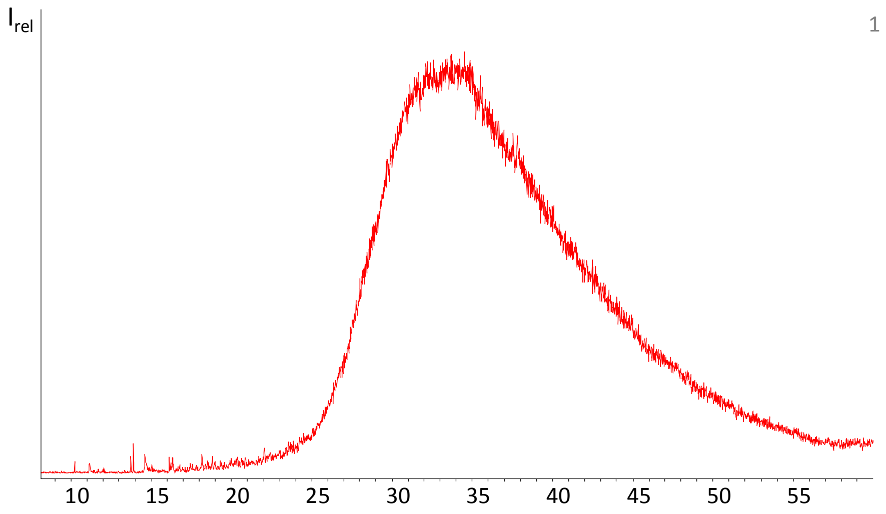

Fig. S1 GC/ECNI-MS full scan chromatogram (*m/z* 50-800; column: OPTIMA 5 MS, 30 m*0.25 mm i.d., 0.25 µm d_f) of sample A-70

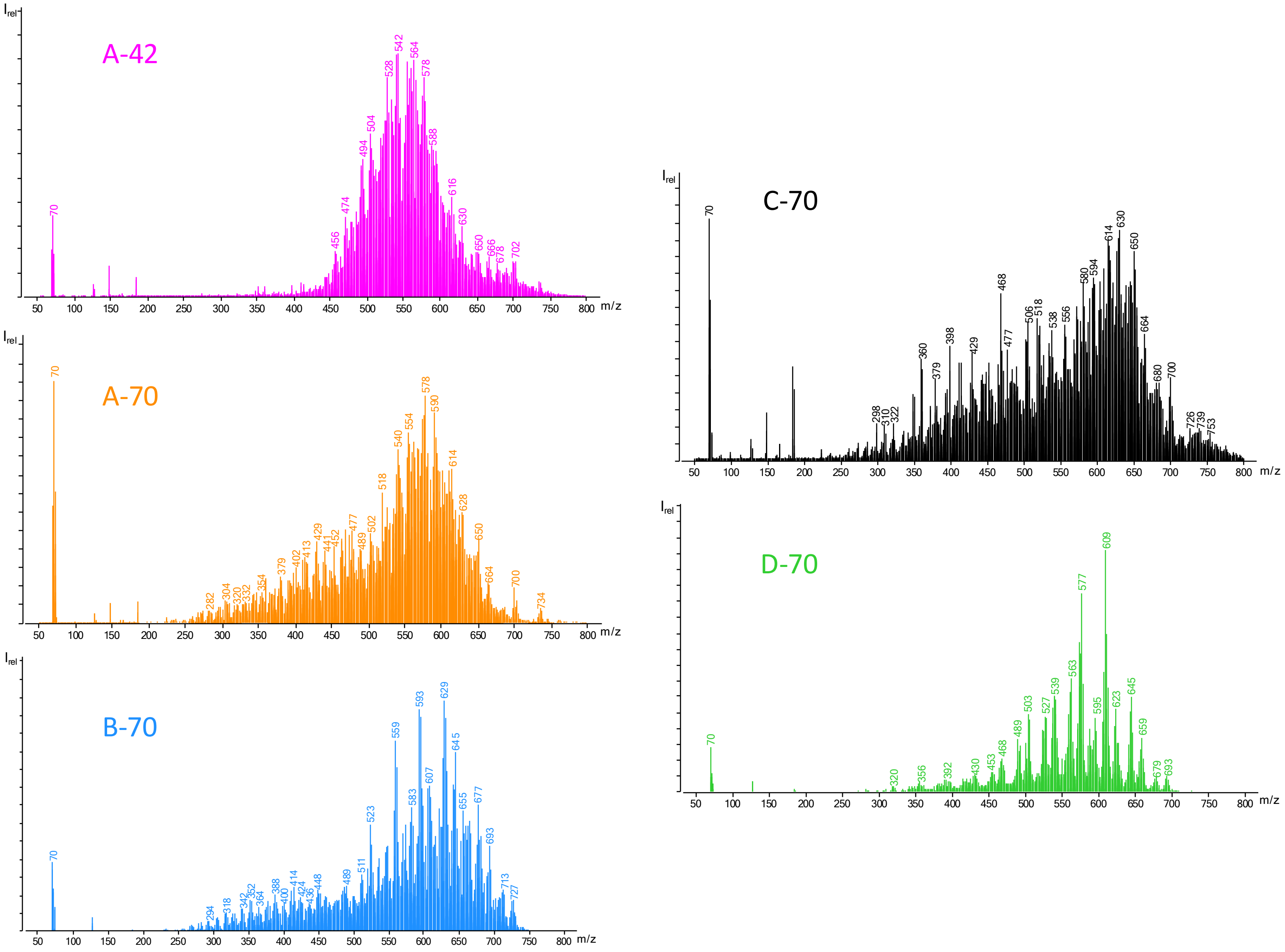

Fig. S2 ECNI mass spectra of samples A-42, A-70, B-70, C-70 and D-70 from 21.2 to 55.8 min (mean value of 2104 scans)

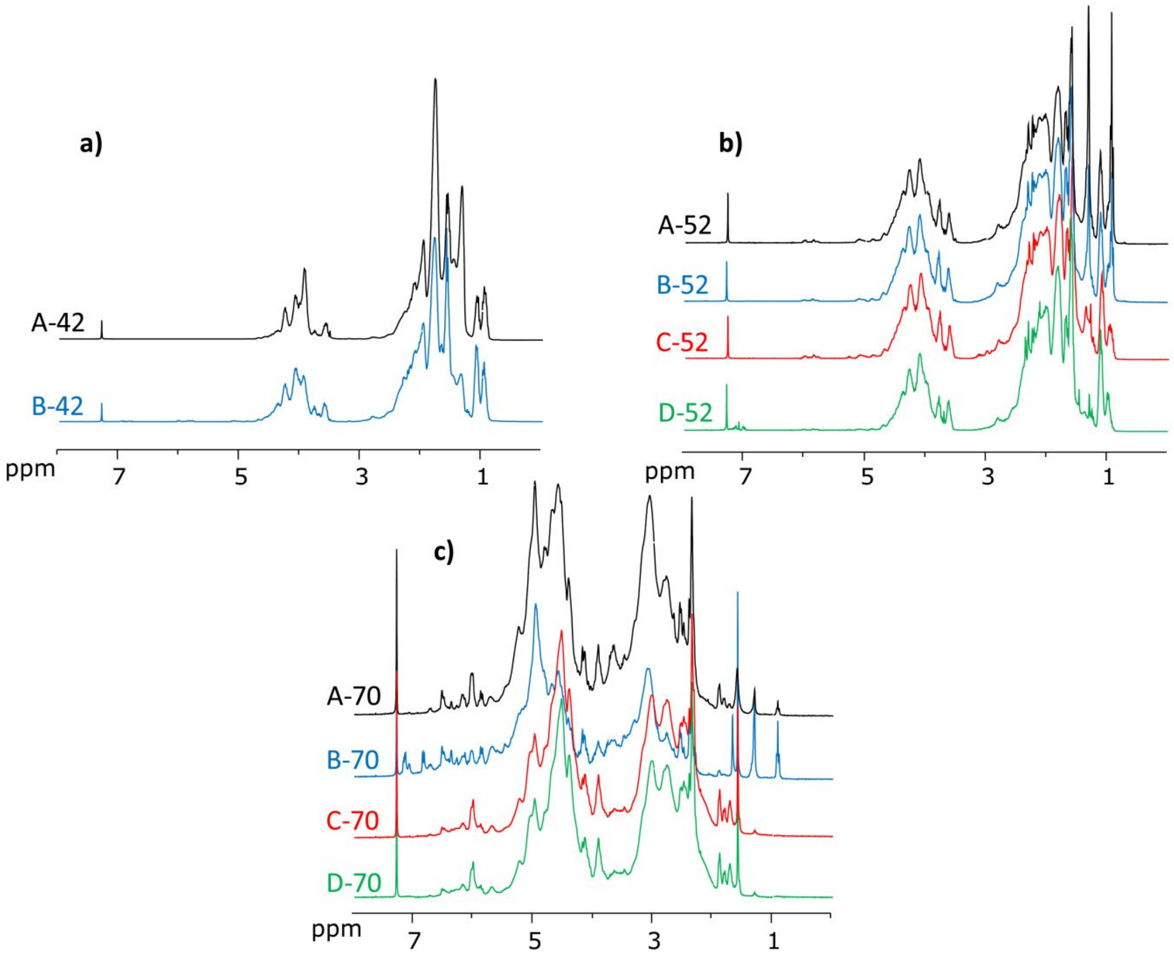

Fig. S3 ^{1}H NMR spectra (300 MHz) of ten technical CP products: **a)** two CP-42, **b)** four CP-52 and **c)** four CP-70 samples were analyzed

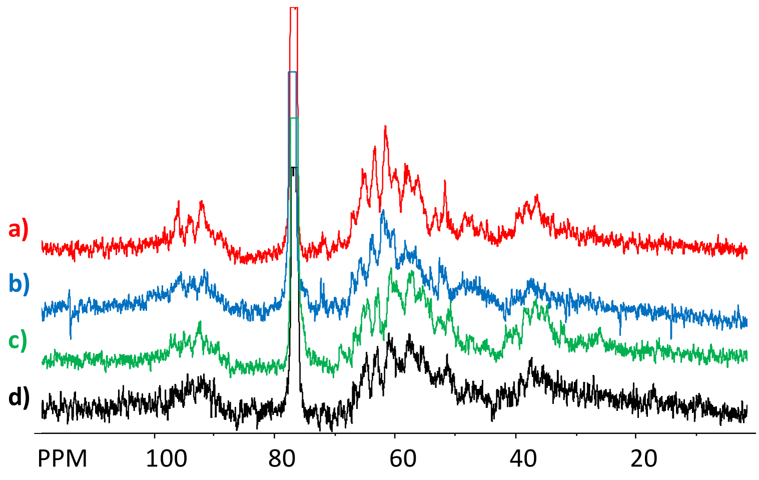

Fig. S4 ^{13}C NMR spectra (300 MHz) of samples **a)** A-70, **b)** B-70, **c)** C-70 and **d)** D-70. At 85-99 ppm, characteristic signals of [-CCl_2-] groups can be observed

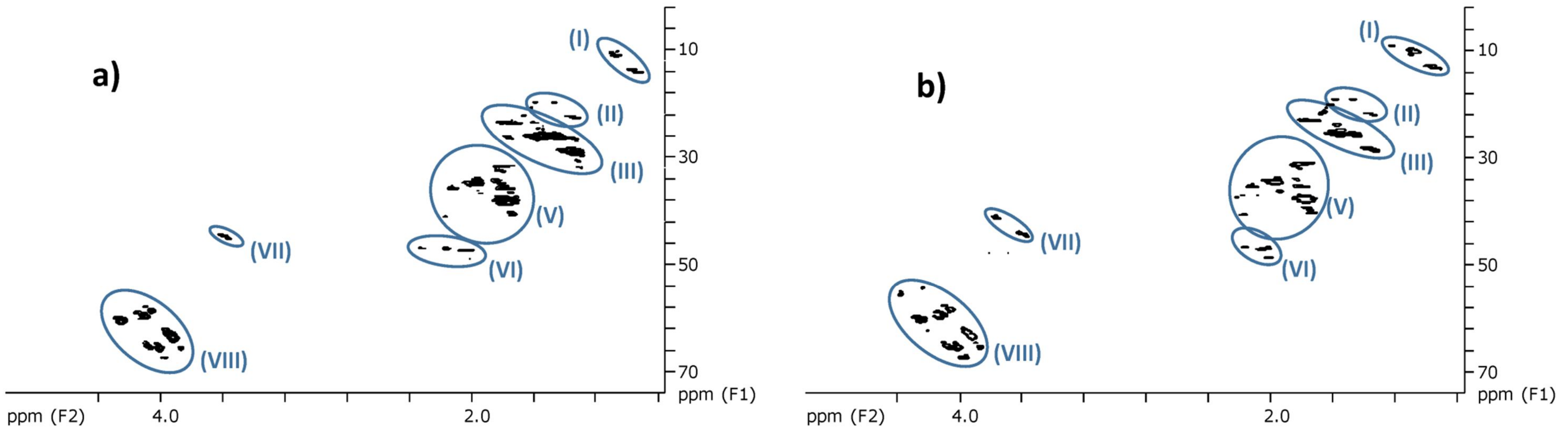

Fig. S5 HSQC spectra (600 MHz) of samples **a)** A-42 and **b)** B-42

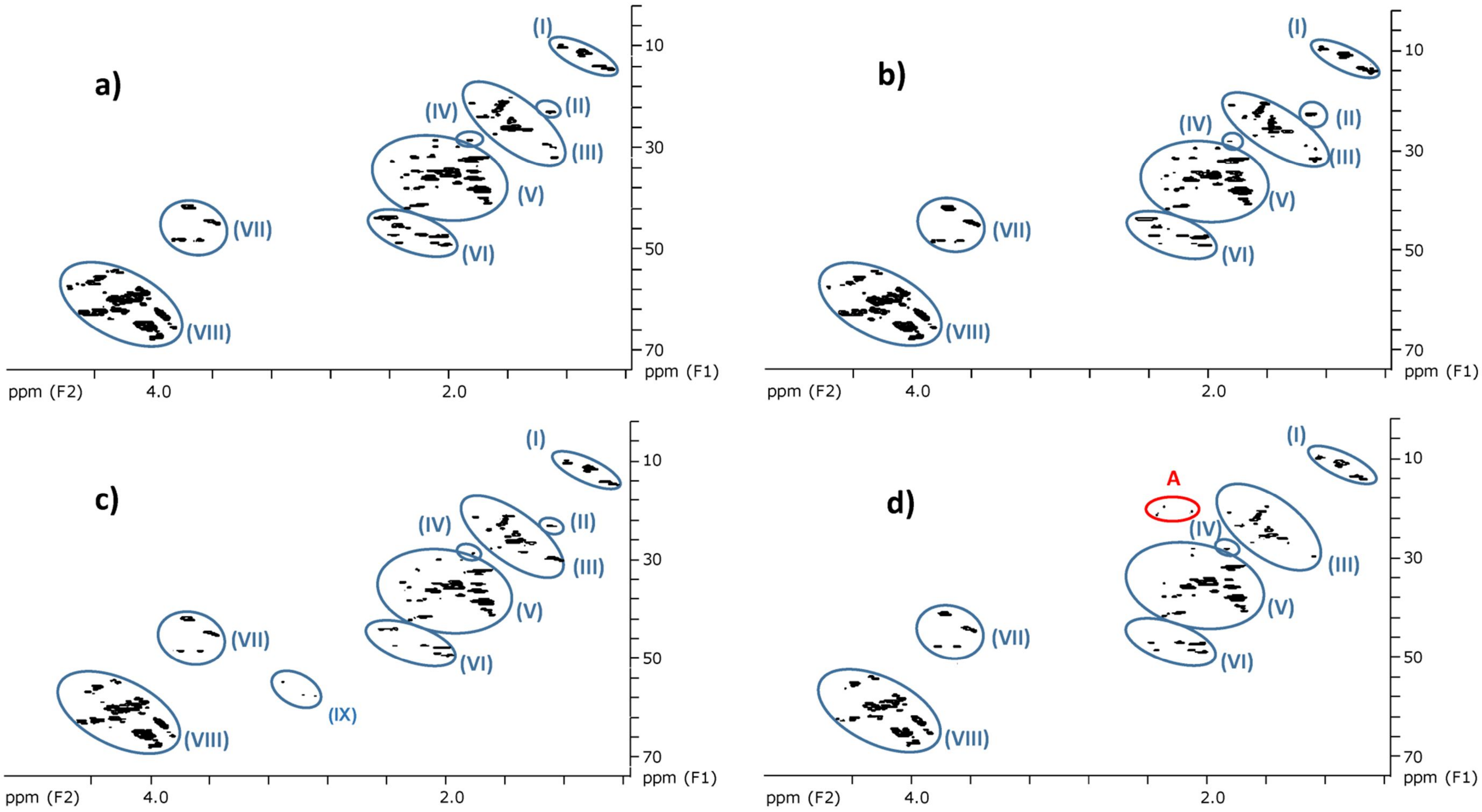

Fig. S6 HSQC spectra (600 MHz) of samples **a)** A-52, **b)** B-52, **c)** C-52 and **d)** D-52

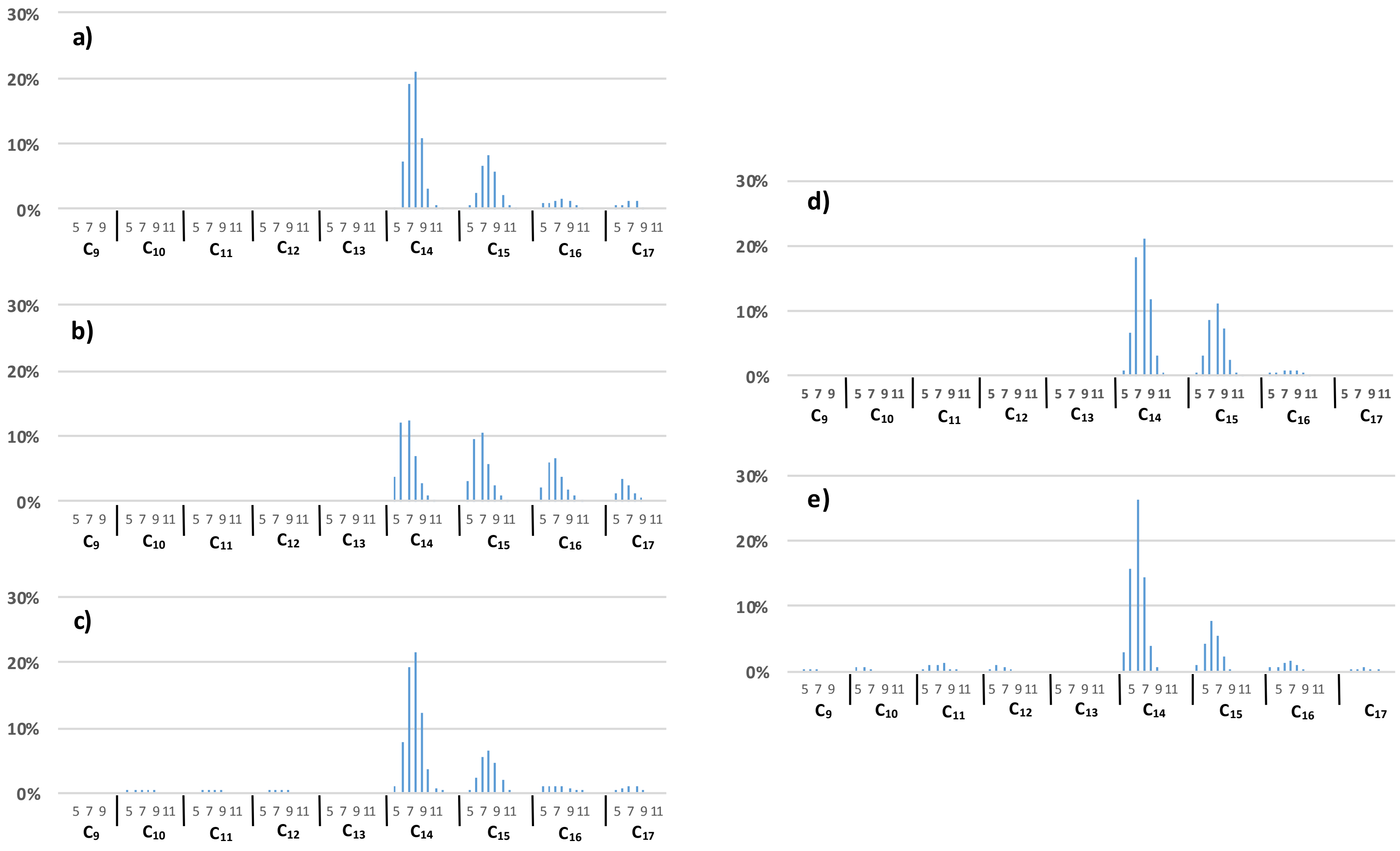

Fig. S7 Homolog patterns of the technical CP mixtures a) A-52, b) B-42, c) B-52, d) C-52 and e) D-52 as determined via GC/ECNI-MS

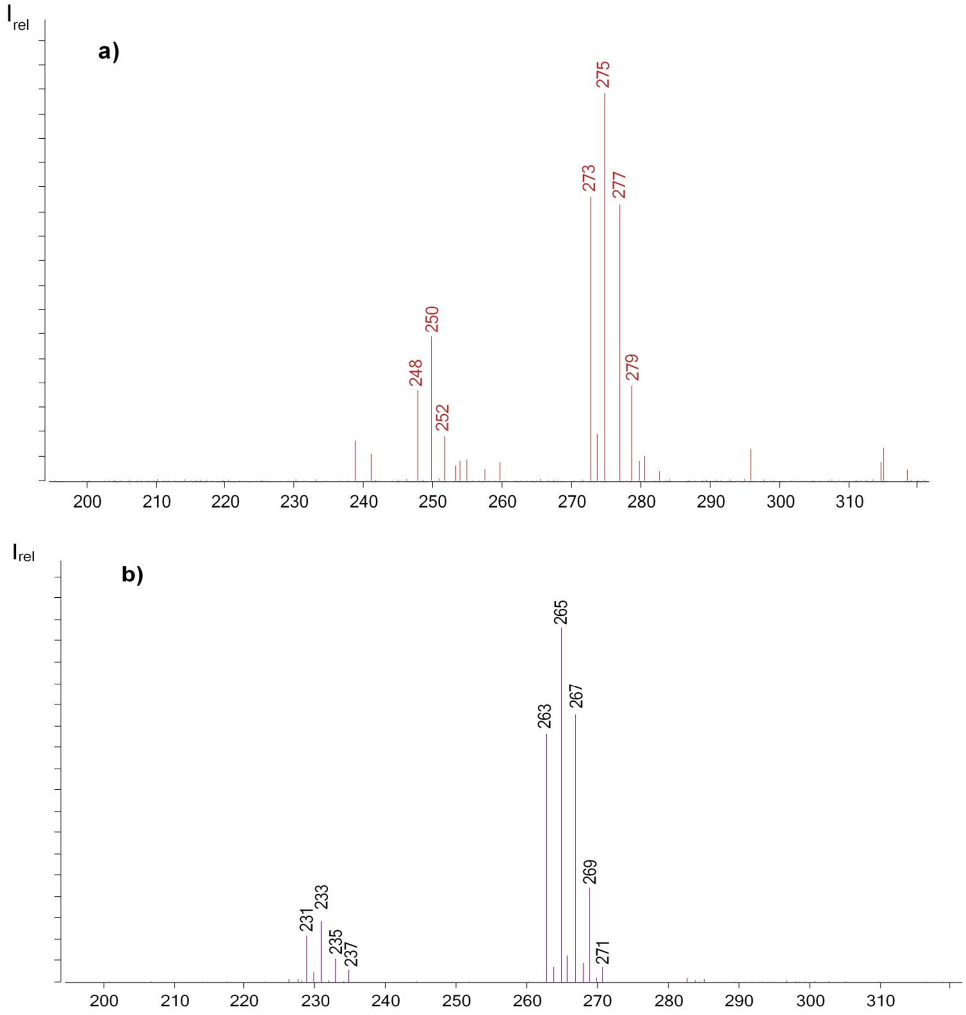

Fig S8 GC/ECNI mass spectra of unknown substances in samples C-70 and D-70, a) substance 1 with a retention time of 17.61 min and b) substance 2 at 18.50 minutes

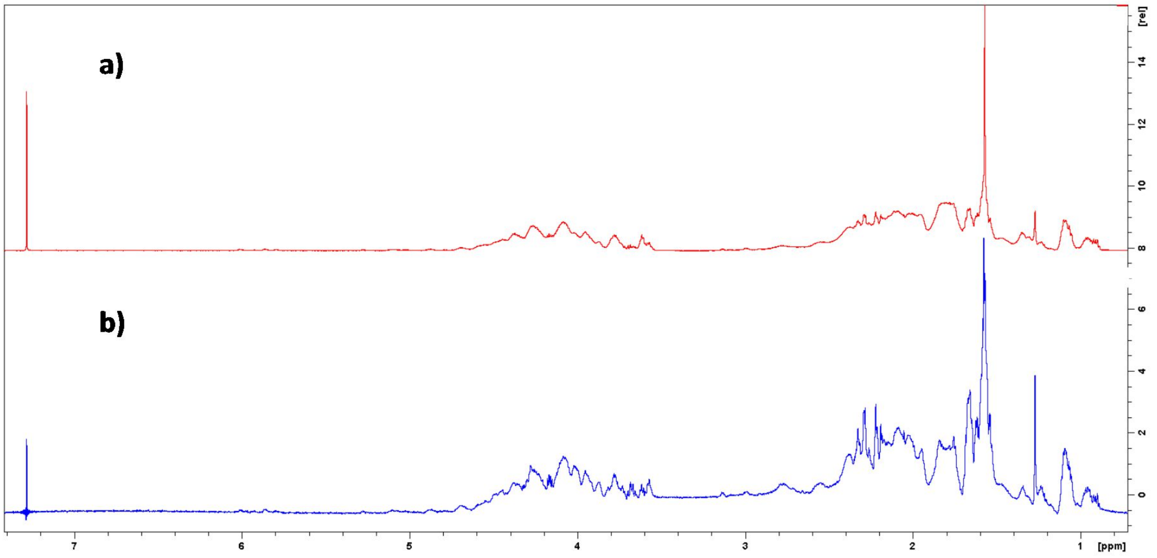

Fig. S9 a) ^{1}H NMR spectrum (600 MHz) and **b)** homonuclear ^{1}H-decoupled ^{1}H NMR spectrum (600 MHz) of sample C-52. NMR parameters were: 5 MHz sweep width, 64 scans, 0.4 s acquisition time. The high number of signals with similar chemical shifts and coupling constants in the highly complex mixture enabled only a partial and insufficient decoupling of the signals. Therefore, the resulting decoupled spectrum appeared almost unchanged and granted no additional insights

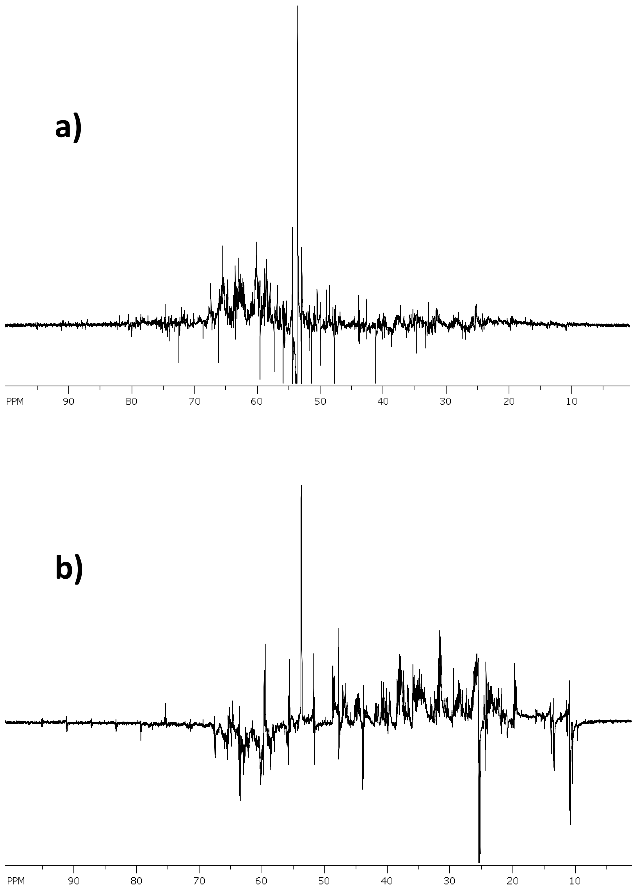

Fig. S10 a) DEPT-90 (150 MHz) and **b)** DEPT-135 (150 MHz) spectra of a self-synthesized C_{11}-CP mixture with 51.2 %Cl. Parameters were: 65.536 data points, 512 scans, sweep width: 36.000 Hz (DEPT-90)/ 24.000 Hz (DEPT-135). The high sample amount (~80 mg) of the mixture needed to generate a response in ^{13}C measurements leads to strongly interfering line broadening, rendering the spectra useless (e.g. no negative peaks should occur in the DEPT-90 spectrum)

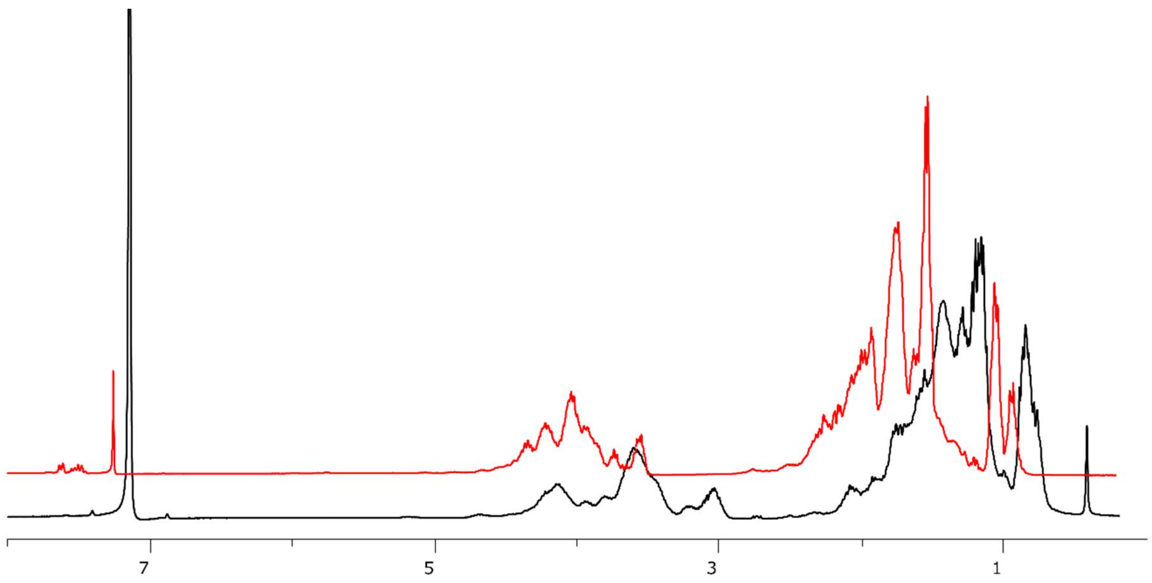

Fig. S11 ^{1}H NMR spectra of a self-synthesized C_{10}-CP mixture (48.6% Cl) in $CDCl_3$ (red) and C_6D_6 (black). The measurement in C_6D_6 stretched the signal shifts over a larger area, while simultaneously showing a stronger line-broadening effect

References

[1] J. Sprengel, W. Vetter, Synthesis and characterization of eight single chain length chlorinated paraffin standards and their use for quantification, Rapid Commun. Mass Spectrom. 33 (2019) 49–56. DOI:10.1002/rcm.8310.

[2] P.B. Pathare, U.L. Opara, F.A.-J. Al-Said, Colour Measurement and Analysis in Fresh and Processed Foods: A Review, Food Bioprocess Technol 6 (2013) 36–60. DOI:10.1007/s11947-012-0867-9.
